# Improvements to the Success of Outbreak Investigations of Legionnaires’ Disease: 40 Years of Testing and Investigation in New York State

**DOI:** 10.1128/AEM.00580-21

**Published:** 2021-07-27

**Authors:** Dianna Schoonmaker-Bopp, Elizabeth Nazarian, David Dziewulski, Ernest Clement, Deborah J. Baker, Michelle C. Dickinson, Amy Saylors, Neculai Codru, Lisa Thompson, Pascal Lapierre, Nellie Dumas, Ronald Limberger, Kimberlee A. Musser

**Affiliations:** a Wadsworth Center, New York State Department of Health, Albany, New York, USA; b Bureau of Water Supply Protection, New York State Department of Health, Albany, New York, USA; c Bureau of Communicable Disease Control, New York State Department of Health, Albany, New York, USA; Centers for Disease Control and Prevention

**Keywords:** *Legionella*, source attribution, outbreaks, testing, collaborative investigation, cutting-edge technologies, outbreak-associated, remediation

## Abstract

Since 1978, the New York State Department of Health’s public health laboratory, Wadsworth Center (WC), in collaboration with epidemiology and environmental partners, has been committed to providing comprehensive public health testing for Legionella in New York. Statewide, clinical case counts have been increasing over time, with the highest numbers identified in 2017 and 2018 (1,022 and 1,426, respectively). Over the course of more than 40 years, the WC Legionella testing program has continuously implemented improved testing methods. The methods utilized have transitioned from solely culture-based methods for organism recovery to development of a suite of reference testing services, including identification and characterization by PCR and pulsed-field gel electrophoresis (PFGE). In the last decade, whole-genome sequencing (WGS) has further refined the ability to link outbreak strains between clinical specimens and environmental samples. Here, we review Legionnaires’ disease outbreak investigations during this time period, including comprehensive testing of both clinical and environmental samples. Between 1978 and 2017, 60 outbreaks involving clinical and environmental isolates with matching PFGE patterns were detected in 49 facilities from the 157 investigations at 146 facilities. However, 97 investigations were not solved due to the lack of clinical or environmental isolates or PFGE matches. We found 69% of patient specimens from New York State (NYS) were outbreak associated, a much higher rate than observed in other published reports. The consistent application of new cutting-edge technologies and environmental regulations has resulted in successful investigations resulting in remediation efforts.

**IMPORTANCE** Legionella, the causative agent of Legionnaires’ disease (LD), can cause severe respiratory illness. In 2018, there were nearly 10,000 cases of LD reported in the United States (https://www.cdc.gov/legionella/fastfacts.html; https://wonder.cdc.gov/nndss/static/2018/annual/2018-table2h.html), with actual incidence believed to be much higher. About 10% of patients with LD will die, and as high as 90% of patients diagnosed will be hospitalized. As Legionella is spread predominantly through engineered building water systems, identifying sources of outbreaks by assessing environmental sources is key to preventing further cases LD.

## INTRODUCTION

Legionella species have been recognized as significant causes of community- and health care-associated respiratory infections. This association was made following the first reported Legionnaires’ disease (LD) outbreak in 1976 (1, 2). Historically, the weak sensitivity of detection methods, the lack of reproducibility, and the fastidious nature of the organism have limited the ability to detect Legionella in patient specimens and environmental sources. Detection is further complicated by the presence of competing flora from both clinical and environmental samples, and semi-selective procedures can enhance Legionella recovery (3).

Testing for Legionella in the Wadsworth Center (WC) laboratory has evolved since microbiological media were first documented for detection of Legionella by culture (4). Initially, due to the unavailability of commercially available, specialized media required for Legionella cultivation in clinical labs, the WC provided the majority of testing for New York State (NYS) clinical specimens, utilizing an in-house media department. Within a few years, media became commercially available, and the WC laboratory transitioned to providing reference services for identification, confirmation, serotyping, and subtyping by pulsed-field gel electrophoresis (PFGE) and whole-genome sequencing (WGS). Outbreak investigation with testing of environmental samples was performed at the request of local or state personnel starting in the 1980s. All isolates were saved either by lyophilization or freezing, resulting in an archived culture collection that includes over 3,000 isolates.

The urinary antigen test (UAT) became widely available by the year 2000 following its development in the late 1980s and 1990s. Reviews of efficacy in the early 2000s demonstrated its usefulness and accuracy as a diagnostic test for L. pneumophila serogroup 1 (SG1) (5). However, UATs are generally not specific for other pathogenic Legionella species and serogroups (3, 6). Despite this new test, culture of patient specimens still remains the gold standard for diagnosis of LD (7).

In 2002, a PCR laboratory-developed test to detect Legionella species and L. pneumophila was implemented (8). The utilization of this assay in conjunction with culture, which was needed to link patient and environmental isolates, both enhanced and streamlined culture efforts. This assay was later improved by being converted into a real-time PCR assay, adding targets to detect L. pneumophila SG1 and an inhibition control. Additionally, a testing protocol, including PCR screening of all clinical specimens and environmental samples prior to culture, was adopted in 2010.

**FIG 1:**
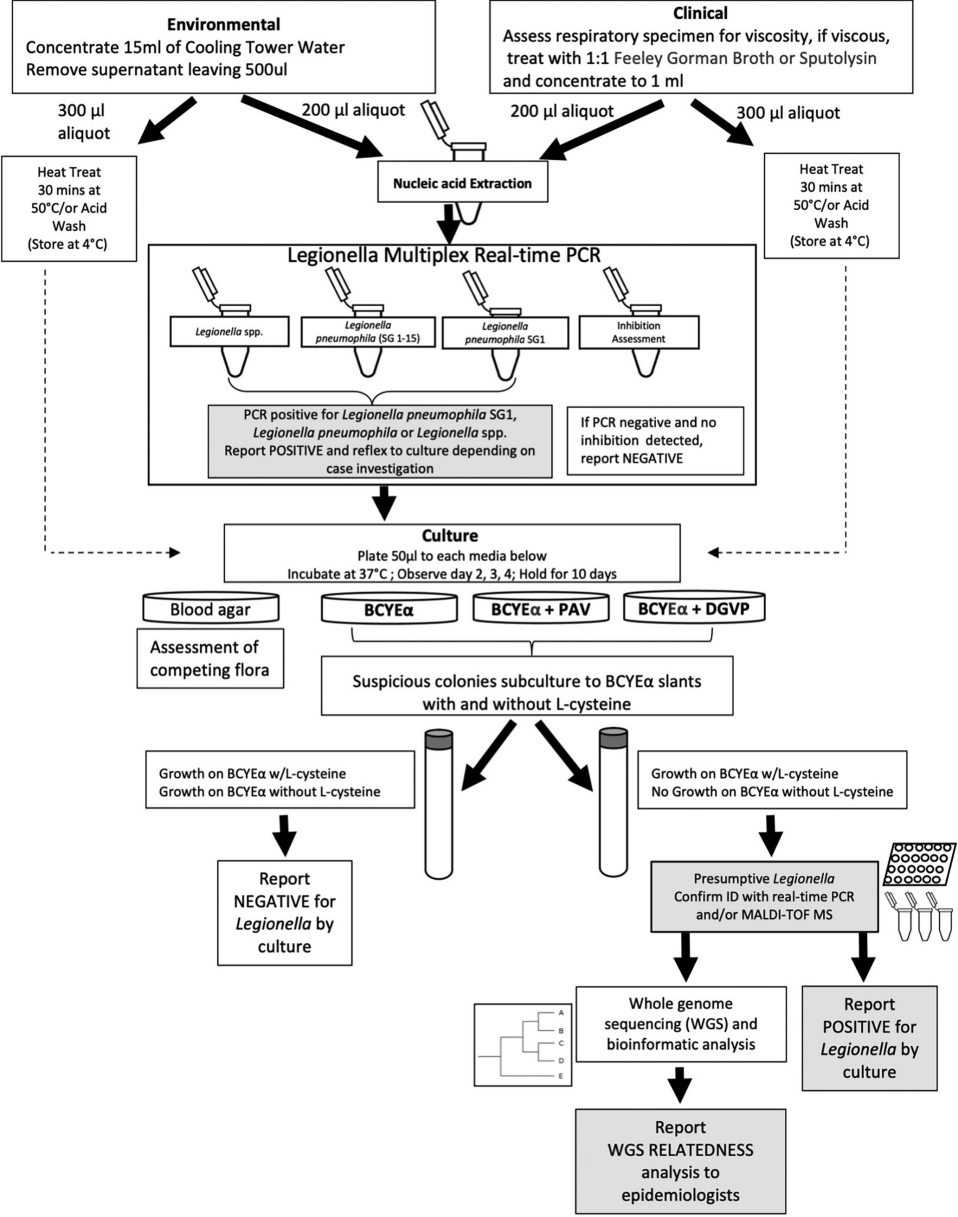
Current *Legionella* testing algorithm for clinical specimens and environmental samples.

In 1990, the need for a method to subtype Legionella species and serogroups led to the application of PFGE for patient and environmental isolates (9). Improvements to the protocol and computerization of the database followed, allowing testing and investigations to be tracked over time. A Centers for Disease Control and Prevention (CDC)-supported contract for whole-genome sequencing (WGS) of Legionella was awarded in 2015, which made WC capable of more improved molecular differentiation among strains. Finally, in 2018, WGS supplanted PFGE for Legionella subtyping. A flow chart describing the current testing algorithm for testing clinical and environmental samples is provided (Fig. 1).

Here, we describe the evolution and application of the testing methods at WC over the past 40 years, including culture, direct fluorescent antibody (DFA) testing, PCR, PFGE, and WGS, as well as discuss the impact of the availability of the UAT. In addition, we describe the importance of collaboration between public health partners along with extensive laboratory testing to determine source attributions that impact the control of this important public health organism.

## RESULTS

### Legionellosis case reports in New York.

**FIG 2:**
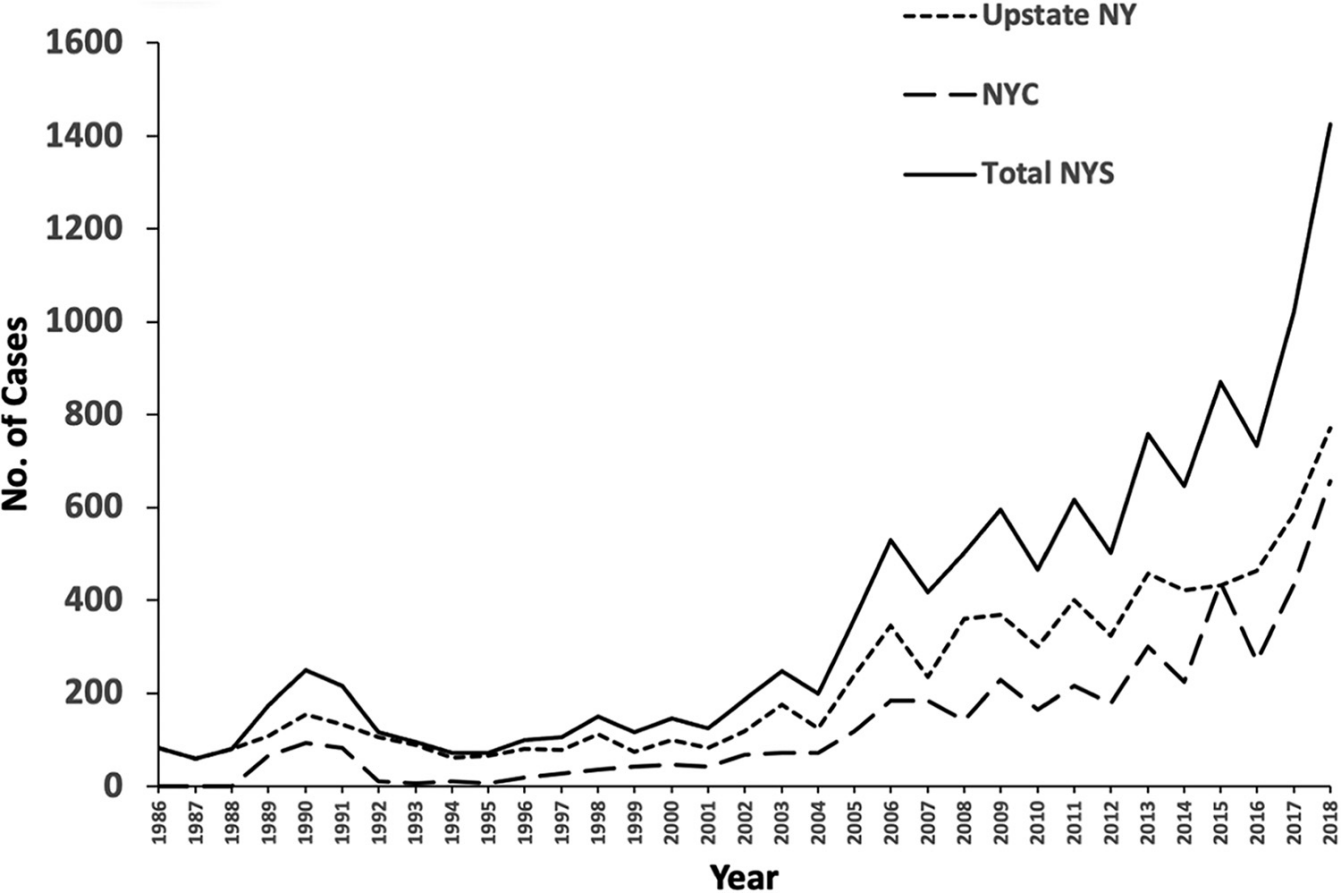
Annual incidence of legionellosis cases recorded through communicable disease reporting by the New York State Department of Health between 1986 and 2018.

Legionellosis cases are reported annually and separately by New York City (NYC; 5 boroughs) and upstate New York. A summary of cases from 1986 to 2018 is provided, demonstrating a substantial increase over this time period from approximately 100 to 1,500 cases per year. Upstate New York cases have historically made up 60 to 65% of statewide cases (Fig. 2).

### Laboratory testing 1979 to 2018.

**FIG 3:**
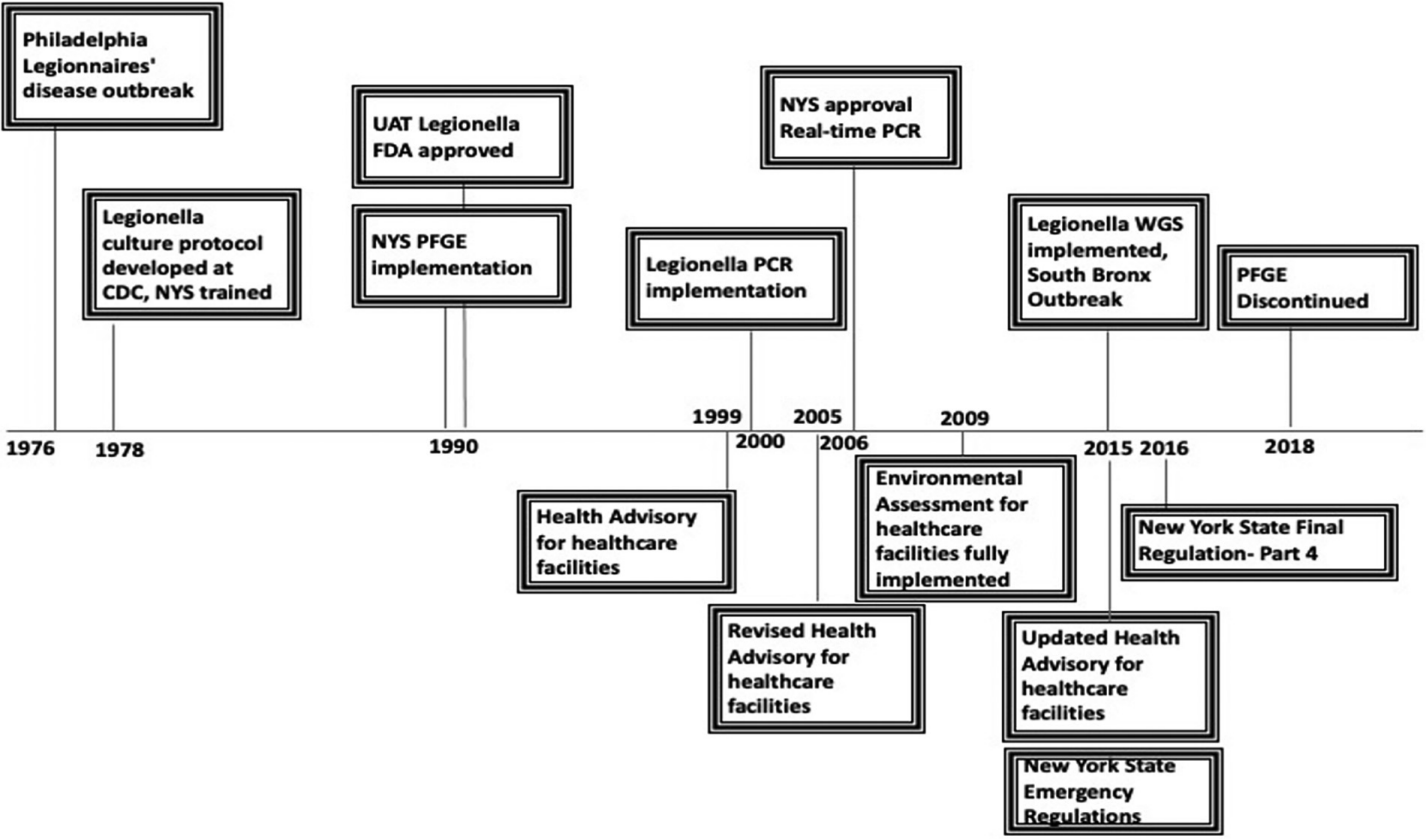
Timeline detailing testing implementation with notable dates on top and advisory and regulation implementation and availability on bottom.

From 1978 to 2018, more than 650 investigation requests were received and supported testing for the investigation of 149 facilities. Laboratory testing between 1979 and 2018 included more than 23,000 clinical specimens and environmental samples that were received for testing with almost 30,000 tests performed as detailed in Table 1 by test method. Changes in testing utilization occurred over time (Fig. 3). Both sera and respiratory specimens were received in the WC laboratory until 1983. Culture of respiratory specimens ranged from 80 to 500 specimens per year. Improvements in culture procedures incorporating additional selective media as well as acid and heat treatments and the addition of PCR screening improved culture positivity over time, as shown in Table 1. PCR results were generally provided <1 day after environmental samples related to clinical cases were collected, allowing for rapid decision-making during investigations. PFGE and, more recently, WGS have been utilized since 1991 to support investigations.

**TABLE 1:** New York State *Legionella* testing, 1979 to 2018

		No. of specimens by test method						
Yr	No. specimens^*a*^	FA (% positive)	IFA^*b*^	Serogrouping	PCR/real-time PCR	Culture^*c*^ (% positive)	PFGE^*d*^	WGS
1979	2,732		2,635			97 (2.1)		
1980	1,553		1,472			81 (4.9)		
1981	2,096		1,975			121 (0.8)		
1982	986		764			222 (8.8)		
1983	1,865		1,588			277 (5.4)		
1984	535	240 (3.3)	295			240 (3.3)		
1985	227	227 (4)				227 (4)		
1986	327	327 (4)				327 (8.9)		
1987	298	298 (1)				298 (4.4)		
1988	386	386 (7.3)				386 (8)		
1989	517	401 (0.5)		85		517 (16.1)		
1990	703	423 (2.1)		104		703 (20)		
1991	553	450 (0.6)		71		553 (12.8)	70	
1992	578			118		578 (20.4)	58	
1993	747			106		747 (14.2)	30	
1994	514			94		514 (18.3)	14	
1995	511			86		511 (16.8)	14	
1996	471			60		471 (12.7)	7	
1997	297			33		297 (11.1)	2	
1998	211			79		211 (29.4)	19	
1999	97			51		97 (52.6)	11	
2000	160			53		160 (32.5)	62	
2001	137			65		137 (47.4)	56	
2002	120			60	16	104 (57.7)	50	
2003	248			85	70	178 (47.8)	55	
2004	82			25	23	82 (67.1)	30	
2005	445			157	178	267 (61)	117	
2006	457			155	165	292 (59.2)	109	
2007	434			207	170	264 (58.3)	90	
2008	481			230	219	262 (49.6)	83	
2009	235				100	135 (51.9)	56	
2010	402				174	228 (67.5)	93	
2011	606				306	300 (38)	106	
2012	344				155	189 (41.3)	64	
2013	185				70	115 (56.5)	60	
2014	264				121	143 (52.4)	85	
2015	932				552	380 (76.8)	278	
2016	486				276	141 (68.1)	88	69
2017	473				263	162 (73.5)	105	53
2018	498				299	199 (66.6)	172	137
Total	23,193	2,752	8,729	1,924	3,157	11,190	1,697	259

a Specimen data from 1979 to 1984 includes both sera and respiratory specimens received; data after 1984 include only respiratory specimens, as sera were submitted to the serology laboratory.

b IFA testing was utilized on acute- and convalescent-phase sera beginning in 1979 and was discontinued in 1984.

c DFA testing was performed in conjunction with culture from 1979 to 1992.

d Abbreviations: DFA, direct fluorescence antibody test; FA, fluorescent antibody test; IFA, indirect fluorescence antibody test; PFGE, pulsed-field gel electrophoresis; WGS, whole-genome sequencing.

### *Legionella* isolate archive.

Between 1982 and 2018, 3,182 Legionella isolates were archived; of these, 1,861 are L. pneumophila SG1, 910 are L. pneumophila of other serogroups, and 411 are other Legionella species (L. anisa, L. bozemanii, L. donaldsonii, L. dumoffii, L. erythra, L. feeleii, L. gormanii, L. gratiana, L. jordanis, L. longbeachae, L. maceachernii, L. micdadei, L. oakridgensis, L. rubrilucens, or L. sainthelensis).

### Legionnaires’ disease outbreak investigations.

As a result of laboratory testing, environmental health assessments, and epidemiologic investigations, the testing performed between 1978 and 2017 supported 157 investigations at 146 facilities. These included outbreaks with multiple Legionella spp. implicated, outbreaks with evidence of persistence (an outbreak with reappearance of the same strain found in previous outbreaks), and outbreaks with evidence of recurrence (an outbreak at a later date caused by a different strain). Many of these investigations provided additional insight into the complexity of Legionella source attribution, persistence, and recurrence in facilities and strategies to improve successful Legionella investigations.

Repeat sampling of the same facilities often extended for many years. The longest WC investigation of collected samples spanned 35 years (Table 2). A total of 60 outbreaks resulted in successful source attributions, as shown in Table 2, with clinical and environmental isolates “matched” having identical PFGE profile patterns. These outbreaks were identified with PFGE-matching patient and environmental isolates and included 36 hospitals, 14 nursing homes, 2 private homes, 2 correctional facilities, 3 residential complexes, 1 hotel, 1 school, and 1 fitness center (Table 2). These 60 outbreaks for which clinical and environmental isolates were identical by PFGE indicate a 38% success rate of identifying a source over time for the 157 WC investigations for which Legionella isolates were available. The identified source for 50 of these 60 outbreaks (83%) was potable water, the source for 8 outbreaks (13%) was a cooling tower, and 2 facilities (3%) had a potable water source and a cooling tower source (Table 2). Most outbreaks (63.3%) were caused by L. pneumophila SG1, while 35.0% were caused by L. pneumophila of other serogroups, and 1.7% were caused by L. micdadei. In 32 of the 60 outbreak investigations (53%), Legionella with a different species, serogroup, or PFGE pattern was also isolated during the investigation in addition to the environmental Legionella matching the clinical isolates (data not shown). Organisms included L. anisa, L. bozemanii, L. dumoffii, L. erythra, L. feelei, L. longbeachae, L. maceachernii, L. rubrilucens, and L. pneumophila serogroups 1, 2, 3, 4, 5, 6, and 12.

**TABLE 2:** Legionnaires’ disease outbreaks with clinical and environmental isolates matched by PFGE (1978 to 2018)

*Legionella*	Facility type	Implicated source	No. (%) outbreaks	Total (%) outbreaks	Collection range (yr[s])	No. matching PFGE over time (yr[s])	Total (%)
LP SG1	Hospital	Potable water	14 (23.3)	17 (28.3)	1–35	5 (7–20)	**38 (63)**
		Cooling tower	1 (1.7)		1–3	1 (3)	
		Both	2 (3.4)		2–7	2 (2–7)	
	Nursing home^*a*^	Potable water	9 (15.0)	12 (20.0)	1–4	2 (2–4)	
		Cooling tower	3 (5.0)		1–10	1 (10)	
	Private home	Potable water	2 (3.3)	9 (15)	1		
	Hotel	Potable water	1 (1.7)		2		
	Correctional facility	Potable water	1 (1.7)		10		
	Miscellaneous	Potable water	4 (6.7)		1–3	1 (3)	
		Cooling tower	1 (1.7)		1		
LP SG3	Hospital	Potable water	3 (5.0)	3 (5.0)	4–22	2 (22)	**21 (35)**
LP SG4	Hospital	Potable water	3 (5.0)	4 (6.7)	1–10		
	Nursing home^*a*^	Potable water	1 (1.7)		1		
LP SG5	Hospital	Potable water	2 (3.3)	3 (5.0)	8–20	1 (8)	
		Cooling tower	1 (1.7)		7		
LP SG6	Hospital	Potable water	5 (8.3)	6 (10.0)	1–25	1 (11)	
		Cooling tower	1 (1.7)		2	1 (2)	
	Nursing home^*a*^	Potable water	1 (1.7)	8 (13.3)	1–2		
	Correctional facility	Potable water	1 (1.7)		11		
LP SG12	Hospital	Potable water	2 (3.3)	3 (5.0)	6–18	1 (6)	
		Cooling tower	1 (1.7)		2	1 (2)	
L. micdadei	Hospital	Potable water	1 (1.7)	1 (1.7)	13	1 (11)	**1 (1.7)**
**Total**				**60 (100.0)**			

a This category includes nursing home and assisted-living facilities.

Investigations from an additional 97 facilities (data not shown) were considered sporadic; they included patient and/or environmental samples associated with correctional facilities (1 facility), hospitals (10 facilities), nursing homes (11 facilities), colleges/schools (1 facility), motels/hotels (4 facilities), county health department investigations (6 facilities), restaurants (1 facility), and others (12 facilities). Laboratory testing of samples from these facilities resulted in the isolation of patient or environmental isolates, but PFGE analysis was of limited utility as no patient and environmental isolates from the same facility were matched. Organisms of 21 different species or serogroups of Legionella were isolated from this testing and archived. In 65 (67%) investigations, a patient isolate was not available, in 19 (20%) investigations, an environmental isolate was not available, and in 13 (13%) investigations, the environmental and patient isolates did not share a matching species, serogroup, or PFGE pattern. Eight (8%) investigations had two or more isolates from patients with matching PFGE patterns, although source attribution could not be demonstrated due to unavailability of an environmental isolate or none with a PFGE match.

### *Legionella* clinical specimen culture isolates.

A review of all clinical specimens with successful culture isolation between 1978 and 2017 revealed that 69.2% were categorized as outbreak associated. These included 352 (81.3%) L. pneumophila SG1 and 81 (19%) either L. pneumophila non-SG 1 or other Legionella species. Overall, during this time period, of all Legionella isolated, 79.4% were L. pneumophila SG1 (Table 3).

**TABLE 3:** Summary of clinical isolates: outbreak-associated versus sporadic, 1978–2017

	No. (%) of isolates		
*Legionella* isolate	Outbreak-associated	Sporadic^*a*^ cases	Total
L. pneumophila SG1	352 (81.3)	145 (75.1)	497 (79.4)
L. pneumophila non-SG 1	63 (14.5)	17 (8.9)	80 (12.8)
Non-L. pneumophila^*b*^	18 (4.2)	31 (16.0)	49 (7.8)
**Total**	**433**^*c*^	**193**	**626**

a Sporadic cases are cases with clinical isolates that do not match other clinical or environmental isolates in the PFGE database.

b Non*-*L. pneumophila isolates included L. anisa, L. bozemanii, L donaldsonii, L. dumoffii, L. feelei, L. gormanii, L. jordanis, L. longbeachae, L. micdadei, and L. oakridgensis.

c 69.2% of all clinical isolates were determined to be outbreak associated.

### Impact of UAT on Legionnaires’ disease outbreak investigations and clinical specimen isolation.

To explore the impact of the UAT on improving or impacting LD outbreak investigations and isolation of Legionella, we divided data into periods before and after the year 2000 (when testing became widely available). For the 60 outbreaks (at 49 facilities) with successful source attributions described in Table 2, we identified 24 outbreaks occurring before 2000 (which included 115 patient isolates) and 36 occurring after 2000 (which included 318 patient isolates) (Table 4). The percentage of outbreaks caused by L. pneumophila SG1 increased from 58.3% to 66.7% after 2000. Outbreaks associated with L. pneumophila non-SG1 or other Legionella species decreased from 37.5% to 33.3% and from 4.2% to 0%, respectively. The total number of Legionella isolated by culture from clinical specimens increased from 140 before 2000 to 486 after 2000. The percentage of clinical isolates identified that were L. pneumophila SG1-positive increased from 65.7% to 83.3% after 2000 (Table 4). The percentage of outbreak-associated clinical isolates identified that were L. pneumophila SG1-positive increased from 67.8% to 86.2% after 2000 (Table 4). Clinical isolates identified as L. pneumophila non-SG1 or other Legionella species decreased from 23.6% to 9.7% and from 10.7% to 7%, respectively. Outbreak-associated clinical isolates identified as L. pneumophila non-SG1 or other Legionella species decreased from 26.1% to 10.3% and from 6.1% to 3.5%, respectively.

**TABLE 4:** Assessing the impact of urinary antigen testing on Legionnaires’ disease outbreaks and *Legionella* isolated from clinical specimens

Outbreaks	Causative agent	No. outbreaks (%) before UAT	No. outbreaks (%) after UAT	Total (%)
Outbreaks^*a*^ from 1978 to 2017, time periods before and after UAT was widely utilized	L. pneumophila serogroup 1	14 (58.3)	24 (66.7)	38 (63.3)
	L. pneumophila serogroup non-01	9 (37.5)	12 (33.3)	21 (35.0)
	Other Legionella species	1 (4.1)	0 (0)	1 (1.7)
	**Total**	**24**	**36**	**60**
All Legionella strains isolated from clinical specimens between 1978 and 2017, time periods before and after UAT was widely utilized	L. pneumophila serogroup 1	92 (65.7)	405 (83.3)	497 (79.4)
	L. pneumophila non-01	33 (23.6)	47 (9.7)	80 (12.8)
	Other Legionella species	15 (10.7)	34 (7.0)	49 (7.8)
	**Total**	**140**	**486**	**626**
Outbreak-associated^*a*^ Legionella strains isolated from clinical specimens between 1978 and 2017, time periods before and after UAT was widely utilized	L. pneumophila serogroup 1	78 (67.8)	274 (86.2)	352 (81.3)
	L. pneumophila non-01	30 (26.1)	33 (10.3)	63 (14.5)
	Other Legionella species	7 (6.1)	11 (3.5)	18 (4.2)
	**Total**	**115**	**318**	**433**

a Legionnaires’ disease outbreaks are defined by clinical and environmental isolates with matching PFGE patterns.

### *Legionella* pulsed-field gel electrophoresis.

PFGE was developed as a fingerprinting tool in 1990 and was initially applied to analyze certain isolates of epidemiological interest. Eventually PFGE was performed on all Legionella isolates in the WC laboratory, providing a robust database of patterns. This testing provided a measure of matched and unmatched determinations for the isolates tested. The resulting PFGE profiles were interpreted based on the criteria described by the CDC, although, in our laboratory, each band difference was considered a different pattern. Matched isolates had identical PFGE profiles, and unmatched isolates had at least one band difference.

The NYS PFGE database contains 1,052 L. pneumophila SG1 isolates representing 357 different PFGE patterns and 301 non-SG1 L. pneumophila with 143 different patterns (Table 5). In addition, 69 PFGE patterns were present among 113 isolates of other Legionella species shown in Table 5, including L. anisa, L. bozemanii, L. donaldsonii, L. dumoffii, L. erythra, L. feelei, L. jordanis, L. gormanii, L. longbeachae, L. maceachernii, L. micdadei, L. oakridgensis, and L. rubrilucens.

**TABLE 5:** PFGE summary of Legionella pneumophila and *Legionella* species, 1990–2018

Legionella serogroup or species	Causative agent^*a*^	Clinical isolates (no. patterns)	Environmental isolates (no. patterns)	Total isolates (% total)
L. pneumophila serogroup	*L. pneumophila* SG1	437 (230)	615 (157)	1052 (78)
	*L. pneumophila* SG2	4 (4)	3 (3)	7 (0.5)
	*L. pneumophila* SG3	4 (4)	16 (9)	20 (1.5)
	*L. pneumophila* SG4	9 (4)	24 (6)	33 (2.4)
	*L. pneumophila* SG4/5	4 (3)	15 (6)	19 (1.4)
	*L. pneumophila* SG5	13 (13)	50 (19)	63 (4.7)
	*L. pneumophila* SG6	15 (13)	97 (44)	112 (8.3)
	*L. pneumophila* SG7	3 (3)	1 (1)	4 (0.3)
	*L. pneumophila* SG8	0	1 (1)	1 (0.1)
	*L. pneumophila* SG10	1 (1)	2 (2)	3 (0.2)
	*L. pneumophila* SG12	8 (4)	27 (12)	35 (2.6)
	*L. pneumophila* SG13	0	1 (1)	1 (0.1)
	Total	498 (279)	852 (261)	1,350
*Legionella* species	*L. anisa*	5 (5)	38 (25)	43 (38)
	*L. anisa/L. bozemanii*	0	4 (2)	4 (3.5)
	*L. bozemanii*	5 (5)	3 (3)	8 (7)
	*L. donaldsonii*	1 (1)	0	1 (1)
	*L. dumoffii*	1 (1)	8 (2)	9 (8)
	*L. erythra*	0	3 (3)	3 (3)
	*L. feelei*	3 (2)	10 (8)	13 (12)
	*L. jordanis*	1 (1)	0	1 (1)
	*L. gormanii*	1 (1)	0	1 (1)
	*L. longbeachae*	1 (1)	2 (2)	3 (3)
	*L. maceachernii*	0	1 (1)	1 (1)
	*L. micdadei*	12 (2)	9 (1)	21 (19)
	*L. oakridgensis*	1 (1)	0	1 (1)
	*L. rubrilucens*	0	4 (2)	4 (7)
	Total	31 (20)	82 (49)	113

a SG, serogroup.

### Impactful *Legionella* investigations.

Over the years, several individual investigations were significant because they furthered the knowledge of LD outbreak source attribution, exposure, persistence, and the application of techniques for investigation of Legionella cases. Table 6 describes some of these investigations that informed Legionella testing and investigation practices as well as identified new sources of Legionella exposure that impacted reducing Legionella exposures in health care facilities. This table lists information on the specifics of each case as well as lessons learned from the investigation.

**TABLE 6:** Summary of impactful Legionnaires’ disease investigations

Location of cases	No. of cases	Yr	Source of exposure	*Legionella* identified	Implications for future Legionnaires’ disease investigations
Small hospital	7	1982	Showers	*L. pneumophila* SG1	Demonstrated showers as a source of exposure, the importance of testing both patients and environmental samples, the usefulness of PFGE testing, and the persistence of *Legionella* in potable water systems (36).
Renal transplant unit large hospital	6	1989	Potable water	*L. pneumophila* SG1,6	First to use PFGE to show that patients and potable water isolates shared patterns that were different from the patterns of other *L. pneumophila* isolates in the facility (33).
COPD^*a*^ patients in a city hospital	5	1990	Potable water used to wash nebulizer	*L. pneumophila* SG3	The PFGE patterns were compared to a neighboring hospital and showed a unique pattern resulting in more understanding of the diversity within *L. pneumophila* serogroup 3. This outbreak also demonstrated the importance of potable water as a source of nosocomial acquisition of *Legionella* (37).
Patient with AIDS	1	1992		*L. pneumophila* SG1	This case provided information of how *Legionella* can persist in a patient over many months even after treatment (38).
New wing of a hospital	2	1992	Nasogastric feeding tubes (tap water)	*L. pneumophila* SG6	These cases lead to change within the hospital to use of sterile water for dilution. An investigation into the plumbing of the facility led to the identification of inadequacies in the plumbing (39).
Transplant patients at a large tertiary care hospital	12	1995–1996		*L. micdadei*	This outbreak highlighted the importance of a heightened suspicion for legionellosis in hospitals that care for immunosuppressed patients and the importance of culture with both selective and non-selective media to detect non-*L. pneumophila* species (40).
Small community hospital	92	1998	Cooling tower	*L. pneumophila* SG1 *L. pneumophila* SG6 in potable water	This large outbreak highlighted the importance of routine maintenance of air conditioning systems and the impact on the patient population (41, 42).
Large hospital	4 2 1	2005 2007 2012	Cooling tower Potable water Cooling tower	*L. pneumophila* SG1,6 *L. pneumophila* SG6,12 *L. pneumophila* SG5 *L. pneumophila* SG1,4,5,6 in cooling tower; SG1,6 in potable water	These investigations demonstrated the complexity of Legionnaires’ disease investigations, the persistence of *Legionella* in the environment, and the need for subtyping and longitudinal testing to solve outbreaks (43).
Adjacent hospital and nursing home	6	2009–2011		*L. pneumophila* SG1,5,6 and *L. anisa*	This investigation demonstrated the efficacy of copper-silver ionization under alkaline water conditions in 2 healthcare facilities (44).
Vulnerable neighborhood in a metropolitan area	138 (16 deaths) 90% hospitalization rate	2015	Cooling tower	*L. pneumophila* SG1	This outbreak led to the enactment of additional regulations to monitor and control *Legionella* growth in potential sources of exposure. WGS was proven to be a useful tool to add discriminatory power above that provided by PFGE to distinguish environmental strains for source attribution in a setting with closely related endemic strains of *Legionella* (19, 20).

a COPD, chronic obstructive pulmonary disease.

## DISCUSSION

Over the past 40 years, improvements and experiences with laboratory testing have impacted WC laboratory testing algorithms (Fig. 1), practices, and outcomes. We have gained a better understanding of the complexity of Legionella in the environment, the importance of extensive patient and environmental testing, the necessity of strain subtyping to determine source attribution, and the persistence or regrowth of Legionella in the environment, even after a risk has been identified and remediation efforts have been put in place.

After a slight increase in legionellosis cases in NYS between 1989 and 1990, the reported number of cases was essentially level for the next decade. Beginning in 2002, an increase occurred, with approximately four times more cases reported in 2017 than in 2003. The increase in the NYS reported cases may be in part due to the introduction of a more robust reporting system as well as improvements in diagnostic methods, such as UAT, and incorporation of PCR into the testing algorithm (13). The reporting system implemented in 2002 is the Electronic Clinical Laboratory Reporting System (ECLRS) (https://www.health.ny.gov/professionals/reportable_diseases/eclrs).

Outbreak investigations involved identification of cases, gathering of epidemiologic information, and patient and environmental testing. Communication between epidemiologists, environmental health scientists, and laboratorians at the local and state levels has been critical. LD is a major concern to public health, and the high case fatality rate of health care-associated LD highlights the importance of case prevention and response activities, including implementation of effective water management programs and timely case identification (7, 14, 15).

The first comprehensive written Legionella guidance was produced by the NYS Department of Health (NYSDOH) in 1999 for distribution to hospitals, nursing homes, and local health departments. It covered case diagnosis and reporting, but it also outlined environmental approaches to the investigation. Those elements included environmental testing, on-site investigation, and control measures. At the time, the consensus was that health care facility water systems should be sampled when there are one or more cases of LD. In the absence of disease, the NYSDOH followed one of two options presented by the CDC (7), that is, to test all patients with health care facility-associated pneumonia and to conduct testing of the hot and cold water systems only if cases were identified. Regular environmental sampling in hematopoietic or solid organ transplant units was recommended.

The guidance section on investigation and control also contained recommendations for short-term treatment and remediation. These included superheating and flushing (water ≥65°C for 5 min) and/or hyperchlorination (≥10 mg/liter of free chlorine) of the water distribution system. Other methods were mentioned, including monochloramine and copper-silver ionization (CSI). The 1999 guidance also contained some elements of operations and maintenance, which included operating heating systems and cooling towers according to manufacturer or industry standards. For potable water systems, this included independently developed protocols for water system control (16).

Updated guidance documents for clinicians, infection control personnel, engineering personnel, and laboratory testing standards were produced in 2005 and 2012 in the form of a Commissioner’s Health Advisory. After 2005, an environmental assessment form was used to evaluate facility vulnerabilities and aid in determining sampling sites (NYSDOH [17]). An additional update to the guidance (NYSDOH [18]) accompanied the promulgation of emergency regulations in 2015 that were made permanent in 2016 (10 NYCRR Part 4 [11]). The latter was in response to a large outbreak in New York City (19, 20) and required establishment of a registry of cooling towers (10 NYCRR subpart 4-1; NYSDOH [21]). Specific requirements for potable water systems in health care facilities were also included in the regulation issued in 2016 (10 NYCRR subpart 4-2 [22]).

Historically, outbreak investigations relied on culture, which can take several weeks to identify and subtype, if successful. The outbreak responses have been impacted and greatly expedited with the use of a real-time PCR-based assay to screen water samples collected from cooling towers or other sources for the presence of Legionella or specifically L. pneumophila SG1. As this PCR assay contains a target for inhibition, it is a valuable screening tool prior to culture, because a negative result indicates culture is not needed. A limitation of its use in outbreak investigation and remediation is the detection of the DNA of nonviable bacteria as well as the inability to discriminate between serotypes other than L. pneumophila SG1. The Legionella real-time PCR assay used at WC has an analytic sensitivity of <1 CFU and a specificity of 100%; many years of evaluation have determined substantial agreement with culture (8). PCR testing for environmental Legionella DNA has provided a significant impact for Legionella investigations in New York by identifying potential sources more rapidly (19, 23).

Through the course of investigations in NYS, source attribution followed by remediation efforts was successful for 60 LD outbreaks using PFGE to match patient and environmental isolates. There were 20 instances of persistence, subsequent additional cases at a facility, which occurred 1 to 20 years after the original outbreak with Legionella isolates with the same PFGE pattern. In total, the 60 outbreaks included 83 clusters of cases with matching environmental and patient isolates in the 49 involved outbreak facilities, as many outbreaks were determined to span multiple years and collections. The complexity of the outbreaks, the persistence or regrowth of Legionella in subsequent years, and the isolation of other Legionella samples in the environment illustrate the difficulty of investigating outbreaks of legionellosis and the need for enhanced testing efforts and longitudinal testing to control outbreaks. It is important to note that testing of multiple patient and environmental isolates related to an investigation can improve the success of source attribution due to the heterogeneous nature of Legionella in environmental sources and the potential for multiple subtypes to be present in infected patients.

The percentage of investigations solved or determined to be an LD outbreak is complicated to conclude and is dependent on the definition of “solved.” If determined by facility, there were 33.6% (49/146) of facilities with both matching environmental and patient isolates that we can conclude are solved. Alternatively, if determined by investigation, 38.2% (60/157) of our investigations were determined to be solved outbreaks. If determined to include recurrence of the same serogroup with different PFGE patterns over time at a facility, we find 41.1% (60/146) to be solved outbreaks. If persistence/regrowth (an outbreak with reappearance of the same strain found in previous outbreaks) is included separately in the calculation, we find 56.8% (83/146) to be solved outbreaks (data not shown).

For the additional 97 investigations not considered “solved” and for which patient and/or environmental testing was also performed, there are a number of reasons for the lack of success. In 67% of these investigations, a patient isolate was not available, possibly due to no testing, early use of antibiotics, or the reliance on nonculture-based testing for Legionella. Eight of these facilities had two or more patient isolates with matching PFGE patterns, which suggests that there may have been a common source, but it was not possible to confirm a specific environmental source. This illustrates that it is important to complete culture-based testing of environmental samples in order to obtain isolates for genetic fingerprinting to determine source attribution. We observed the presence of multiple Legionella species and serogroups in 42.3% of all facility investigations (data not shown), demonstrating the complexity of determining the source of cases, the importance of thorough testing algorithms, and the importance of directing effective control or eradication efforts.

Notably we found 69.2% of Legionella patient isolates to be outbreak associated (Table 3) compared with 30.8% of isolates that were found to be sporadic. This percentage is much higher than some reported rates of 4 to 11% (6, 24–26) and may be attributed to our testing algorithms, investigative approaches, and regulatory actions. Our long-term data indicate that Legionella of other species and serogroups is less commonly isolated from clinical specimens.

Analyzing data from before and after the year 2000 when the UAT became commonly utilized (Table 4), we observed an increase in the percentage of outbreaks caused by L. pneumophila SG1 (58.3% to 66.7%) as well as an increase in the percentage of L. pneumophila SG1 isolated from clinical specimens among isolations in that time period (65.7% to 83.3%). This increase is likely due to the reliance on the UAT as a clinical diagnostic tool. In their 2015 review, Mercante and Winchell state that disease attributed to L. pneumophila of other serogroups and Legionella species decreased by 79% in the United States between 1980 and 1998 (3), suggesting that L. pneumophila SG1 is overrepresented in current estimates of LD, and that, while the UAT has been a valuable tool, reliance on this one diagnostic test may result in significant numbers of undetected LD cases (5, 6, 25, 27). Our data do appear to agree with this statement, but may be impacted by practices, testing, and investigations in our jurisdiction.

The increase in the percentage of L. pneumophila SG1 isolated was almost 18% higher between the time periods and more prominent than the 2.2% increase for L. pneumophila of other serogroups (5.3% to 7.5%) or the 3.0% increase of Legionella of other species (2.4% to 5.4%; Table 4). The percentage of outbreaks caused by L. pneumophila SG1 is lower than frequently reported, which also may be due to one or more of the following: aggressive investigation of cases, a high degree of clinical suspicion, public health follow-up on cases, and the prioritization of environmental assessment and testing.

PFGE has been very useful in source attribution for LD outbreaks, demonstrating useful diversity of patterns. There was pattern diversity among both the patient and environmental isolates, suggesting an extensive subset of organisms responsible for disease. This diversity illustrates the importance of strain discrimination to determine the source of outbreaks. WGS has further refined the ability to discriminate specific disease-causing organisms from the background flora in the environment. During the large NYC community LD outbreak associated with a cooling tower in 2015 that included 138 patients and 16 deaths, we found the Legionella isolates examined to be highly conserved, and WGS proved to be a powerful and useful tool to discriminate among isolates where PFGE could not (20). New advances in WGS and other next-generation technologies continue to provide improvement to the detection, characterization, and relatedness of patient specimens and sources that may one day make culture isolation obsolete.

Many of the investigations described in Table 6 highlighted the ability of Legionella to persist in a location, sometimes even after remedial measures had been taken, and underscored the need for longitudinal testing and archiving over years to solve some outbreaks. Other investigations demonstrated the practicality and importance of PFGE and, later, WGS in Legionella testing for identifying the source of an outbreak or demonstrating relatedness between clinical isolates. Some investigations aided in updating and perfecting sanitation and maintenance efforts in facilities. Throughout the years, more information on the diversity and persistence of Legionella has been acquired as a result of case and outbreak investigations.

In summary, the testing and investigation methods utilized in New York State over the past 40 years have changed and improved over time, and lessons have been learned from investigations. Continued efforts to utilize advanced testing and to prioritize investigational approaches have led to a high level of success for both solved outbreaks and culture isolation of clinical isolates. This highly successful program, particularly for linking clinical and environmental isolates, has impacted Legionella investigations in our state. The importance of environmental, epidemiological, and laboratory collaboration to determine outbreak association and impact the control of this important public health problem is critical to our successful efforts for legionellosis investigations in NYS.

## MATERIALS AND METHODS

### Specimens and culture.

Clinical specimens submitted from laboratories and environmental samples were collected from local facilities by state personnel as part of investigations of suspected, probable, or confirmed legionellosis cases. As indicated in Fig. 1, clinical respiratory specimens and environmental samples were plated on buffered charcoal yeast extract (BCYE) agar, BCYE agar with added anisomycin, polymyxin B and vancomycin (prior to 2012, BCYE agar with added anisomycin, polymyxin B, and cefamandole was used), and blood agar plates (28). Environmental water samples were concentrated 30-fold by centrifugation, and 50-μl aliquots were plated onto the culture medium and incubated at 37°C. Typical Legionella colonies were subcultured to BCYE with and without l-cysteine. Biochemical tests, including autofluorescence, catalase, oxidase, urease, gelatinase, and browning on Feeley Gorman agar (29, 30), were performed. A step of acid washing with 0.2 mol/liter HCl-KCl, pH 2.2, for 3 min (12, 31) or heat treatment at 50°C for 10 min (32) and an additional plating on BCYE medium with glycine, vancomycin, polymyxin B, and dyes were used for more difficult samples in which bacterial overgrowth was found.

### Direct and indirect fluorescence antibody testing.

Indirect fluorescence antibody (IFA) testing was applied to acute- and convalescent-phase sera beginning in 1979 (1) but was unable to detect cases in a timely and sensitive manner and was discontinued entirely in 1999. Between 1979 and 1984, the titer for L. pneumophila was detected by a combination of two tests: sera were screened using a fluorometer and the titer was determined using a conventional IFA test, which eventually transitioned to only providing the titration of sera. Direct fluorescence antibody (DFA) testing was applied to clinical specimens and environmental samples beginning in 1979, but, as weaknesses in specificity and sensitivity became more apparent, the test was no longer used as a routine assay on raw specimens in 1992. Culture isolates were identified to the serogroup or species level by DFA tests until 2017 (M-tech, Atlanta, GA). Typical Legionella colonies that were subcultured to BCYE with and without l-cysteine were identified to the serogroup or species level by DFA tests (M-tech, Atlanta, GA).

### PCR detection of *Legionella* in isolates, clinical specimens, and environmental samples.

Nucleic acid extraction was performed with the Epicentre Technologies MasterPure complete DNA purification kit (Madison, WI) according to the manufacturer’s instructions. Each extraction incorporated negative and positive extraction controls. PCR and, later, real-time PCR assays were developed and validated for use over time both for initial testing of specimens and samples to rapidly screen samples for prioritizing culture efforts and to confirm bacterial isolates following culture. The most recent assay for clinical specimens detects and differentiates Legionella spp. L. pneumophila and L. pneumophila SG1 and uses an internal control to assess for inhibitory substances in the sample. The *wzm* gene, which specifically detects L. pneumophila SG1, the *mip* gene, which detects L. pneumophila serogroups 1 to 15, and the 23S rRNA gene, which detects all Legionella spp. and serogroups (8, 10), were used. The real-time PCR assay to confirm bacterial isolates also detects L. anisa and L. micdadei and does not include an inhibition control.

### Matrix-assisted laser desorption/ionization time-of-flight mass spectrometry.

After cultivation, isolates were tested to confirm species-level identification by matrix-assisted laser desorption/ionization time-of-flight mass spectrometry (MALDI-TOF MS) according to the manufacturer’s instructions and as described by others (9).

### Pulsed-field gel electrophoresis.

DNA was prepared from Legionella isolates harvested from BCYE plates after 24 to 48 h and digested with 50 U of SfiI (Roche, NEB). Subsequent modifications were made after the original publication (33) so that the procedure was shorter and was similar to the standard PulseNet protocol for DNA preparation (34). PFGE conditions consisted of 6 V/cm with an initial switch time of 7 s and a final switch time of 70 s. PFGE patterns were analyzed using BioNumerics (Applied Maths, Austin, TX). The resulting PFGE profiles were interpreted based on the criteria described by Tenover et al. (35), although, in our laboratory, each band difference was considered a different pattern.

### Whole-genome sequencing and bioinformatic analysis.

Nucleic acid extraction for both clinical and environmental isolates was performed using a modified EpiCentre DNA extraction procedure. DNA sequencing was carried out using the Illumina MiSeq platform (Illumina, San Diego, CA, USA) as described previously (17, 18, 45).

Raw reads were mapped to a reference strain, and single-nucleotide polymorphisms (SNPs) were called by using Samtools/BCFtools; a minimum of Q20 for mapping quality and base-call quality, 10× minimum depth, and 95% of allele read agreements were used. SNP alignments were built, and pairwise number of polymorphic site differences were counted and used to determine the likelihood that two or more samples were related. In general, SNP differences can vary between investigations, but less than 20 SNP differences between isolates are considered related.

### Epidemiological investigation.

In the early years following the 1976 outbreak, a confirmed case of LD was defined as a patient who had radiographic evidence of pneumonia in addition to either isolation of L. pneumophila from respiratory secretions or a greater than or equal to 4-fold rise of serum antibodies to L. pneumophila, as measured by indirect immunofluorescence. The case definition was later modified to include DNA detected by PCR and urine antigen test (UAT) when those tests became available. When guidance documents became available from the CDC or the Council for State and Territorial Epidemiologists (CSTE), the case definitions were amended to reflect current practice. In order to be classified as a nosocomial case, a patient had to have been hospitalized for at least 48 h before the onset of respiratory symptoms or readmitted to the hospital within 10 days of a previous discharge. For outbreak investigations, epidemiologic information was gathered, environmental samples were tested, and subtyping was performed to aid in identifying the source of exposure for cases.

The CDC defines a Legionnaires’ disease outbreak as “two or more cases associated with the same possible source during a 12-month period” (https://www.cdc.gov/legionella/outbreaks.html). For this analysis, we have included cases with the same PFGE pattern as part of the same outbreak even if they occurred more than 1 year later after the original outbreak. If a different species, serogroup, or PFGE type was responsible for subsequent cases, it was counted as a different outbreak.

### Environmental health investigation and water sampling.

The New York State Department of Health (NYSDOH) program for response to legionellosis has been in place since the disease became reportable in 1985. During an investigation, the sample locations are selected and, if available, compared with any historic data, including history of positive culture. Analyses have included Legionella culture, Cu, Ag, pH, conductivity, temperature, and chlorine residual. Culture monitoring, to determine the effectiveness of any remediation, has relied on determining the number of Legionella-positive sites (percent positivity) to follow the persistence or control of the Legionella population. All hot water samples were first draw samples. Samples of 100 ml were aseptically collected after faucet aerators were removed. Sample bottles contained thiosulfate to inactivate free chlorine and other oxidants. Samples were capped and stored on synthetic ice bricks (0°C to 4°C) in coolers and then transported to the laboratory.

**Data availability.** Newly determined assembled genome sequences were deposited at NCBI with the BioProject ID PRJNA725843.
